# The Association Between Cognitive Domains and Postural Balance among Healthy Older Adults: A Systematic Review of Literature and Meta-Analysis

**DOI:** 10.1007/s11910-023-01305-y

**Published:** 2023-10-19

**Authors:** Nahid Divandari, Marie-Louise Bird, Mahdi Vakili, Shapour Jaberzadeh

**Affiliations:** 1 Monash Neuromodulation Research Unit, Department of Physiotherapy, School of Primary and Allied Health Care, Faculty of Medicine, Nursing and Health Science, Monash University, PO Box 527, Melbourne, Frankston, VIC 3199 Australia; 2 School of Health Sciences, University of Tasmania, Newnham Tasmania 7248, Launceston, Australia; 3 Mowbray Medical Clinic, Invermay, TAS Australia

**Keywords:** Global cognition, Executive function, Processing speed, Relationship, Physical mobility, Static vs dynamic balance

## Abstract

**Purpose of Review:**

This review aims to explore which cognitive domain is more closely associated with which type of balance (static or dynamic).

**Resent Finding:**

Based on recent reviews, inhibitory control, a part of cognition, plays a crucial role in balance performance. Previous reviews report significant links between cognition, mobility, and physical function in older adults. However, evidence regarding the relationship between cognition and balance scores remains inconclusive.

**Summary:**

The strength of association between cognition and balance appears to be domain-specific and task-specific. Executive function exhibits the strongest correlation with balance, while episodic memory shows a small link with dynamic balance. Processing speed and global cognition demonstrate moderate correlations. Additionally, there is a slight association between cognitive domains and static balance. Further research is needed to elucidate the underlying mechanisms and develop targeted interventions for managing balance-related concerns that are domain-specific and task-specific.

**Supplementary Information:**

The online version contains supplementary material available at 10.1007/s11910-023-01305-y.

## Introduction

Population ageing is a global issue [1], with one-third of those over 65 years old falling each year [2]. A major contributing factor to these falls is impaired balance, defined as difficulty in keeping the center of gravity within the base of support [3]. Balance is the complex integration and coordination of several underlying systems that cover sensory/perceptual processes, cognitive influences, and motor performance [4]. This sensory cognitive–motor network ensures the precision of movements [5]. Recent studies have shown an association between cognition and balance in older adults.

Cognition includes multiple domains that work together to process information during tasks [6] such as balance [7]. Cognition helps to have an effective adaptation to changing environments [8]. It includes domains such as executive function [9], processing speed, memory, attention, and language [10]. However, not all domains of cognition are equally correlated with physical function [11].Ageing does not homogeneously affect all cognitive domains [10]. Moreover, mobility is more strongly related to fluid aspects of cognition [11]. Therefore, it seems that some cognitive domains have a stronger association with balance than others.

Static balance entails maintaining stability while remaining stationary, whereas dynamic balance requires maintaining stability while moving. These different demands may require different cognitive processes. The impact of cognitive processes on motor skills, such as postural balance, depends on task difficulty [12, 13•]. The dynamic balance task is more challenging, requiring greater mental processing capacity [14••]. Therefore, balance and cognition may be more closely related to dynamic tasks than static ones. Comparing the associations between cognition and static versus dynamic balance can help us to understand these differences.

The relationship between cognitive domains and both static and dynamic balance tasks is poorly understood. A 2020 review showed a clear association between physical and executive function, but the link between executive function and balance was less certain due to limited evidence [15••]. They included seven studies examining the association of executive function and balance, and a few of them included people with mild cognitive impairment in their review. In a 2022 review, inhibitory control (a subdomain of cognition) was highlighted as crucial for balance task performance [7], but their results were limited to just inhibitory control. No review studies have looked at the relationship between different cognitive domains and balance tasks specifically. A meta-analysis in 2016 focused on the association between some cognitive domains and balance [16•]; however, it was limited to only five articles and did not compare this association between static and dynamic balance tasks. Further examination of the recent existing literature determines which cognitive domains are most strongly associated with each type of balance task. This may help prioritize identifying the type of cognitive domain that may be added as a dual task activity to balance intervention to enhance their effectiveness. This can lead to improved rehabilitation outcomes and more effective screening and diagnosis of cognitive and balance problems.

To fill this gap in the literature, a systematic review and meta-analysis were conducted to compare the association of various cognitive domains with static and dynamic balance in older community-dwelling adults. To our knowledge, this study is the first to compare the correlation between cognitive domains and both dynamic and static balance tasks. To check the genuine relationship between balance and cognition, we concentrated on single tasks. The decline in dual-task performance in older adults can result from either cognitive or physical changes associated with ageing. Furthermore, since dual-task conditions involve cognitive components, examining the relationships between balance and cognitive tasks would lead to problems with collinearity. This makes it challenging to determine whether any observed correlations are due to shared cognitive components or a genuine relationship between balance and cognition [16•]. The aims of this review are: 1. to check the evidence for associations between cognitive domains and balance among healthy older adults, 2. to investigate whether cognitive domains vary in their correlation with dynamic and static balance measures, and 3. to investigate whether this association is different from dynamic balance compared to static balance among different cognitive domains.

## Methods

### Literature Search

#### Data Sources and Search Strategy

The review was conducted according to the Preferred Reporting Items for Systematic Reviews and Meta-Analysis (PRISMA) [17]. All studies that examined the association between balance and cognitive function in healthy adults over 60 years of age until the end of May 2023 were included. Studies were searched online using electronic databases, including EMBASE, MEDLINE, Scopus, PubMed, Science Direct, and Ovid. In addition, the reference lists of existing studies and reviews were searched manually. The search terms were postural stability OR postural sway OR balance OR mobility OR equilibrium OR physical function AND cognition OR cognitive domains OR attention OR executive function OR processing speed OR memory OR language AND association OR correlation OR relationship. Where appropriate, the keywords were modified based on the glossary of each database and mapped to Medical Subject Heading (MeSH) terms. Appendix A (in supplementary documents (SD)) provides an example of the search strategy for the EMBASE database that has been provided. The results were exported to Endnote X9 (Clarivate Analytics, Philadelphia, USA) to remove duplicates.

#### Study Selection

Two reviewers (authors N.D and M.V) independently screened titles and abstracts to ensure they met inclusion criteria. The full articles were read by two authors (authors N.D and Sh.J), discussed and compared with inclusion criteria. Any disagreements were resolved via consultation with a third reviewer (author M.B) if required.

The inclusion criteria were as follows: 1. English-language papers, published in peer-reviewed journals. 2. Investigated dynamic or static balance. 3. Investigated the cognitive ability by tests of global cognition or tests for any specific cognitive domain. 4. Cross-sectional studies investigated the association between balance and cognitive domains based on concurrent collection of data in a single task. 5. Healthy adults older than 60 years without any neurological pathological conditions.

The exclusion criteria were as follows: 1. Any pathological conditions, such as dementia and its subtypes, or any cognitive impairment. 2. Participants with neurological pathological conditions such as stroke, Parkinson’s disease, or traumatic brain injury. 3. Used self-reports as the outcome measure of balance (e.g., the Balance Self-Perception Test).

### Quality Assessment and Data Extraction

The quality of the selected studies was assessed by two reviewers (authors N.D and Sh.J). The Newcastle–Ottawa Scale, adapted for cross-sectional studies, was used for the assessment of quality. The Newcastle–Ottawa Quality Assessment Scale includes eight multiple-choice questions from three broad domains: four items related to the selection of cohorts, one item related to the comparability of cohorts, and three items related to the assessment of outcomes [18]. The risk of bias was assessed using an adapted version of the AXIS-tool using two reviewers (authors N.D and Sh.J) [19]. Disagreements were discussed and resolved via the third person (author M.B).

Data were extracted, categorized, and entered into a spreadsheet, and then verified by another reviewer (author Sh.J). Regular meetings between the two reviewers were held weekly during the data extraction and analysis to achieve consistency and consensus (author N.D and Sh.J). For each included study, the following details were extracted (Tables 1, 2 and Table 1 in SD): demographic information (sample size, sex, and mean age), cognitive domains (global cognition, executive function, memory, processing speed, attention, and language), outcome measures for balance (Score on Berg Balance Test, Time of stance in different foot positions, Timed Up and Go Test, Distance on Functional Reach Test, Equilibrium score based on postural sway, Postural sway, Score on Tinetti Balance Test, Fullerton Advanced Balance (FAB) score and stability index) and the results (significant or insignificant results and Pearson correlation). All the extracted information was categorized based on the cognitive domains and balance tests used in the studies (Tables 1, 2 and Table 1 in SD).

**Table 1:** Characteristics of the relationship between measures of executive function and dynamic/static balance

First Author	Number of participants	Mean Age	% Female	Balance Task	Executive Function	Association
**Executive Function and dynamic balance: EF**						
**Kang, et al. **[29]** 2022**	94	72.6 ± 5.3	100%	TUG	Seoul Neuropsychological Screening Battery	NS r: 0.099
Jovanovic, et al. [30] 2022	98	68.5	83.6%	TUG	Trail Making Test	S r: 0.217
**Matos, et al. **[31]** 2020**	28	66.7 ± 7.6	84%	TUG	N-Back Test	S r: 0.531
** Netz, et al. **[32]** 2018**	33 M	77.2 ± 5.5	0%	TUG	MOXO DNSCPT ADHD Test, based on Go No Go Test	S 0.653
Kose, et al. [33] 2016	80	75.7 ± 5.8	45%	TUG	Trail Making Test B	S r: 0.358
**Blackwood, et al. **[34]** 2015**	47	74.9 ± 5.9	48.6%	TUG	Trail Making Test B	S r: 0.308
**Kawagoe, et al. **[35]** 2015**	32	73.1	37.5%	TUG	N-Back Test	S r: 0.58
Berryman, et al. [36] 2013	48	70.5 ± 5.3	58%	TUG	Stroop Test	S r: 0.565
**Herman, et al. **[37]** 2011**	265	76.4	58%	TUG	Verbal Fluency	S r: 0.217
Hirato, et al. [38] 2010	493	73.3	66.7%	TUG	∆Trail Making Test	S r: 0.335
**Won, et al. **[39]** 2014**	164	66 ± 4.6	66.5%	FRT	Clock Drawing Test	S r: 0.201
Tsutsumimato, et al. [40] 2013	59	88 ± 87	83%	FRT	Trail Making Test	S r: 0.10
**Redfern, et al. **[41]** 2019**	34	76 ± 4	61.7%	Postural Sway	Task Switching Test	NS r: 0.29
**Redfern, et al. **[42]** 2009**	24	74.2 ± 4.4	50%	Postural Sway	MAPIT battery for Motor Inhibition	S r: 0.39
Van Iresel, et al. [43] 2008	100	80.6	50%	Postural Sway	Trail Making Test	S r: 0.893
Rabbit, et al.[44] 2006	69	73.2 ± 8.1	57.97%	TBT	Color/Word Stroop Test 1	S r: 0.326
**Zettel-Watson, et al. **[45]** 2017**	50	69.5 ± 8.1	64%	FABS	Summing Stroop Color-Word Test, BP distraction, and EPT	S 0.31
Muir-Hunter, et al. [46] 2014	24	76.18	100%	BBS	Trail Making Test A	S r: 0.550
**Executive Function and static balance**						
**Redfern, et al. **[41]** 2019**	34	76 ± 4	61.7%	Postural sway	Perceptual and Motor Inhibition Test	S r: 0.54
**Netz, et al. **[32]** 2018**	38 F	77.2 ± 5.5	100%	Postural sway	MOXO DNSCPT ADHD Test, based on Go No Go	S r: 0.427
Muir-Hunter, et al. [46] 2014	24	76.18	100%	Postural sway	Trail Making Test A	S r: 0.089
**Redfern, et al. **[42]** 2009**	24	74.2 ± 4.4	50%	Postural sway	MAPIT battery for Motor Inhibition	NS r: 0.21
Boolani, et al. [47] 2019	11	76.55 ± 7.58	72%	mCTCIB	Serial subtraction 7	S r: 0.433
Demnitz, et al. [48] 2017	387	69.0 ± 5.1	19%	SLS	Digit span	NS r: 0.056
**Won, et al. **[39]** 2014**	164	66 ± 4.6	66.5%	SLS	Clock Drawing Test	S r: 0.07
Tsutsumimato, et al. [40] 2013	59	88 ± 87	83%	SLS	Trail Making Test	S r: 0.36
Bruce- Keller, et al. [49] 2012	50	74.2 ± 7.8	42%	Stance time on SPPB	Digit Symbol Test	S r: 0.07
Hirato, et al. [38] 2010	493	73.8	66.7%	SLS	∆Trail Making Test	S r: 0.312
Rosano, et al.[50] 2005	2893	73.4 ± 2.8	52%	Stance time	Trail Making Test	S r: 0.190

*No* Number of participants, *M* male, *F* Female, *Number* reference of the study, *TUG* Timed Up and Go Test, *FRT* Functional Reach Test, *TBT* Tinetti Balance Test, *BBT* Berg Balance Test, *FABS* Fullerton Advanced Balance Score, *SPBB* Balance Score on the Short Physical Performance Battery, *SLS* Single leg stance time, *mCTCIB*, Modified Clinical Test of Sensory Interaction on Balance, *NS* Non-significant, *S* Significant. *r* correlation. Bolds are studies which had MMSE score > 24 as inclusion criteria

**Table 2:** Characteristics of the relationship between measures of global cognition and dynamic/static balance

First Author	Number of participants	Mean age	% Female	Balance task	Global cognition	Association
**Global cognition and dynamic balance**						
Zhao, et al. [51] 2022	107	71.7 ± 5	70%	TUG	Mini-Mental State Examination	NS r: 0.21
Jovanovic, et al. [30] 2022	98	68.5	83.6%	TUG	Montreal Cognitive Assessment	NS r: 0.125
Abe, et al. [52] 2017	169	72.4 ± 4.8	47.3%	TUG	5-Cog Battery	S r: 0.371
Kose, et al. [33] 2016	80	75.7 ± 5.8	45%	TUG	Mini-Mental State Examination	NS 0.126
Kwan, et al. [53] 2011	280	74.9 ± 6.4	42.8%	TUG	Mini-Mental State Examination	NS r: 0.30
**Won et al. **[39]** 2014**	164	66 ± 4.6	66.5%	FRT	Mini-Mental State Examination	NS 0.168
Tsutsumimato, et al. [40] 2013	59	88 ± 87	83%	FRT	Mini-Mental State Examination	NS r: 0.07
Woo, et al. [54] 2017	385	79.1 ± 2.9	64%	BBS	Mini-Mental State Examination	S r: 0.485
Muir-Hunter, et al. [46] 2016	24	76.18	100%	FABS	Montreal Cognitive Assessment	S r: 0.510
**Global cognition and static balance**						
Imaoka, et al. [55] 2022	20	70.4 ± 4.9	45%	Postural sway	Montreal Cognitive Assessment	NS r: 0.35
**Goto, et al. **[56]** 2018**	79 M	67.8 ± 5	0%	Postural Sway	Mini-Mental State Examination	S r: 0.239
Muir-Hunter, et al. [46] 2016	24	76.18	100%	Postural Sway	Montreal Cognitive Assessment	S r: 0.510
**Won et al. **[39]** 2014**	164	66 ± 4.6	66.5%	Postural Sway	Mini-Mental State Examination	NS 0.022
Zhao, et al. [51] 2022	107	71.7 ± 5	70%	SLS	Mini-Mental State Examination	NS r: 0.08
Abe, et al. [52] 2017	169	72.4 ± 4.8	47.3%	SLS	5-Cog Battery	S r: 0.338
Tsutsumimato et al. [40] 2013	59	88 ± 87	83%	SLS	Mini-Mental State Examination	S r: 0.19
Bruce- Keller, et al. [49] 2012	50	74.2 ± 7.8	42%	Balance SPPB	Mini-Mental State Examination	NS r: 0.20
Rosano, et al. [50] 2005	2893	73.6	52%	SLS ratio	Mini-Mental State Examination	S r: 0.17

*No* Number of participants, *M* male, *F* Female, *Number* reference of the study, *TUG* Timed Up and Go Test, *FRT* Functional Reach Test, *TBT* Tinetti Balance Test, *BBT* Berg Balance Test, *FABS* Fullerton Advanced Balance Score, *SPBB* Balance Score on the Short Physical Performance Battery. *SLS* Single leg stance time, *mCTCIB* Modified Clinical Test of Sensory Interaction on Balance. *NS* Non-significant, *S* Significant, *R* correlation. Bolds are studies which had MMSE score > 24 as inclusion criteria

### Data Synthesis and Analysis

Meta-analyses were conducted using Comprehensive Meta-Analysis software, version 4. The effect size index was calculated. Pearson’s r coefficient reported in the included studies was used [20]. If any study reported Spearman’s rho or beta coefficient, it was converted to Pearson’s r coefficient by using the following formula: Spearman’s rho was transformed using the equation (r = 2sin [rs π/6]) [21]. Beta coefficients were transformed into Pearson’s correlation coefficients. The formula is: r = 0.98β + 0.05γ (if (β ≥ 0, γ = 1; β < 0, γ = 0) [20, 22]. To interpret the results, pooled rz values were retransformed to r values with an inverse Fisher z transformation: r = e2rz − 1 / e2rz + 1, where e is approximately equal to 2.718 and rz is the Fisher-z-transformed r value [23]. Effect sizes were categorized based on static and dynamic balance outcome measures and cognitive domains. Due to differences in the study sample and design, the random-effects model was used to calculate the pooled mean effect size [16•, 23]. Q-statistics were used to test the heterogeneity across studies [24]. The I^2^ index was used to test consistency between them [25]. The I^2^ index ranging from 0 to 100%. A percentage of 25%, 50%, and 75% is assigned to low, moderate, and high levels of heterogeneity, respectively [25]. Forest plots with 95% confidence intervals (CIs) are reported and standardized effect sizes were interpreted as small (0.1), medium (0.3), or large (0.5) [26]. A leave-one-out sensitivity analysis was conducted to identify studies contributing excessively to heterogeneity. The association of cognition with each balance task was checked to assess certainty (or confidence) in the body of evidence for an outcome.

If better performance in balance tests was associated with better performance on cognitive tests, the association was considered positive, even if it was reported as a negative association in the study. For example, some studies have shown a negative association between the time of the TUG test and the number of correct answers on cognitive tests. This means that better balance (shorter time for the TUG test) was associated with better cognitive results (higher scores for correct answers to cognitive tests). Therefore, in this case, the association is reversed, and considered positive in this review [16•].

## Results

### Studies and Participants

**Fig. 1:**
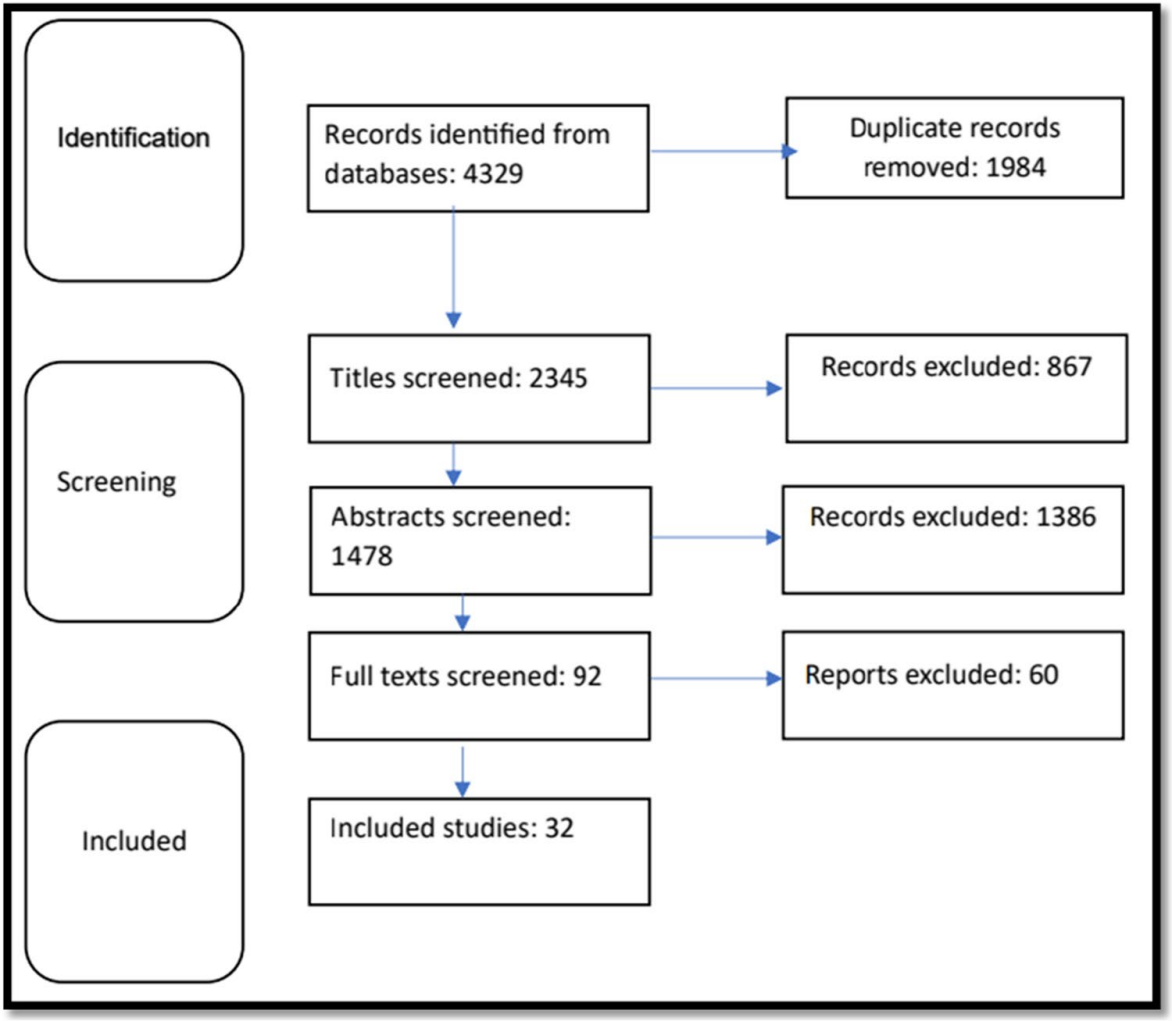
Flowchart for the process of literature search

After removing duplicates and screening titles and abstracts, 92 studies were identified. After applying the eligibility criteria, only 32 studies met the inclusion criteria and were finally included in this review (Fig. 1).

Balance association with global cognition, executive function, processing speed, and episodic memory were reported among studies. Global cognition was analyzed in 13 studies, executive function in 22 studies, processing speed in nine studies, and episodic memory in seven studies. A few authors have not reported a correlation when the association was not significant. All were contacted via email. The characteristics of the included studies and reported correlations are summarized in Tables 1, 2 and Table 1 in SD.

Cognitive domains and balance tests were classified based on the descriptions provided in each study. If the name of those was not specified in a study, that was classified based on a systematic review about clinical tests of balance used in seniors and recent articles about domains of cognition and their assessments [27, 28]. Most commonly, the outcome measure for cognition, dynamic balance, and static balance were executive function, TUG, and a single-leg stance and postural sway, respectively.

Some of the studies reported that their participants had a score of higher than 24 in the Mini-Mental State Test (MMSE). These studies are summarized in bold in Tables 1, 2 and Table 1 in Supplementary documents. The results of the systematic review for each cognitive domain are summarized as follows:

#### The Systematic Review of The Association Between Cognitive Domains and Balance

##### The Association Between Executive Function and Balance

Eighteen studies investigated the relationship between executive function and dynamic balance. The most commonly used measure for dynamic balance was the Timed Up and Go (TUG) test time, employed in ten studies. Postural sway, Functional Reach Test (FRT), Berg Balance Test (BBT), Turn 360, and Fullerton Advanced Balance Scale (FABS) were used in the remaining studies. All but two studies reported a significant association between executive function and dynamic balance, with effect sizes ranging from small to moderate (Table 1).

Eleven studies examined the association between executive function and static balance. The main outcome measure was stance time, particularly standing on one leg in six studies, followed by postural sway in four studies. With the exception of two studies, most reported a significant association between executive function and static balance, albeit with mostly small effect sizes. Overall, the results indicated that better executive function was associated with better dynamic and static balance (Table 1).

##### The Association Between Episodic Memory and  Balance

Seven studies investigated the association between processing speed and dynamic balance. Four studies examined this relationship with static balance. Various outcome measures for dynamic balance were used, while postural sway and single leg stance time were chosen outcome measures for static balance. Significant associations were reported in nearly all the included studies. The results showed that faster processing speeds were associated with better dynamic and static balance (Table 1 in SD).

##### The Association Between Episodic Memory and Balance

Eight studies were focused on exploring the connection between measures of episodic memory and balance. Out of these studies, six specifically investigated the relationship between episodic memory and dynamic balance. However, the majority of the studies did not find a significant association between episodic memory and balance (Table 1 in SD).

##### The Association Between Global Cognition and Balance

Eighteen studies examined the relationship between global cognition and balance. Out of these studies, nine specifically focused on investigating the association between global cognition and dynamic balance. The findings from these studies are mixed, as some suggest a non-significant association between global cognition and both static and dynamic balance, while others indicate a significant association between the two variables (Table 2).

#### Meta-analysis for Assessing the Associations Between Cognitive Domains and Balance

##### The Effect Size for the Correlation of Executive Function and Balance

**Fig. 2:**
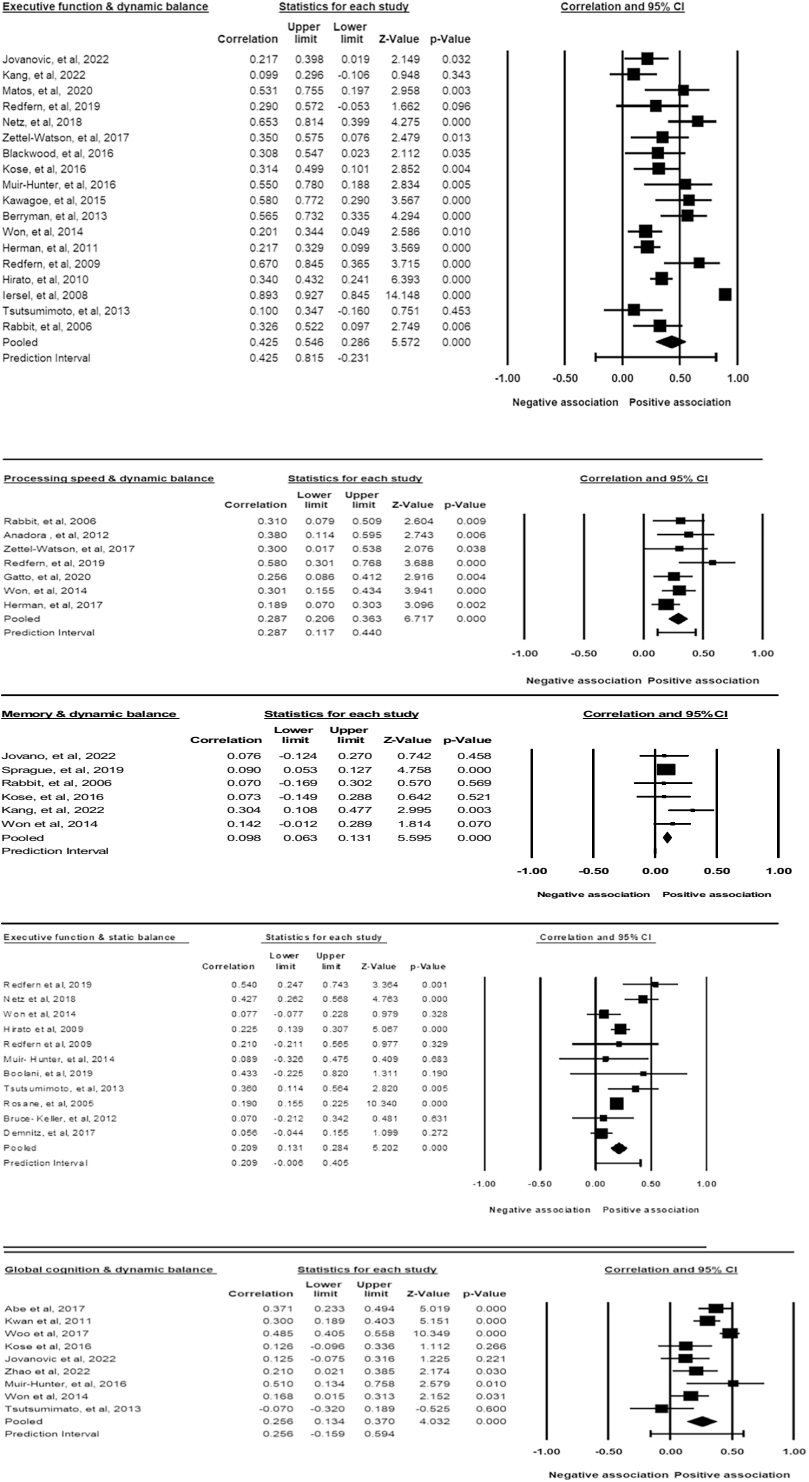
Statistical summary and forest plot of effect sizes for the association of executive function, processing speed, global cognition, and memory with dynamic balance

A meta-analysis including 18 studies revealed a medium effect size of 0.425 (95% CI = 0.286–0.546, *p* = 0.000; Fig. 2) in favor of a positive association between executive function and dynamic balance. The results suggest that older adults with higher executive function scores performed better on dynamic tests. However, the studies were substantially heterogeneous (Q = 151.216, p = 0.000, I^2^ = 88%). The result is stable after removing the studies one by one.

**Fig. 3:**
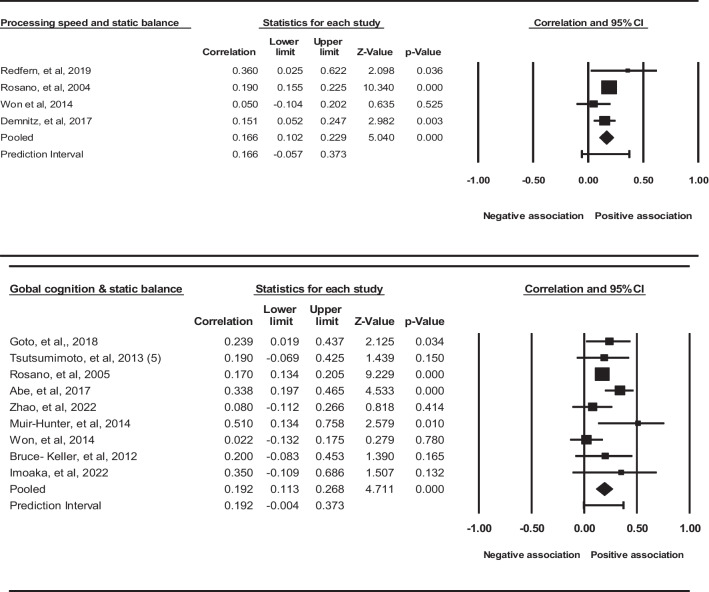
Statistical summary and forest plot of effect sizes for the association of executive function, processing speed, and global cognition with static balance

A meta-analysis of 11 studies revealed a small effect size of 0.209 (95% CI = 0.131–0.284, *p* = 0.000; Fig. 3) in favor of a positive association between executive function and static balance. These results suggest that older adults with higher executive function scores performed better on static balance tasks. However, the studies were substantially heterogeneous (Q = 26.192, *p* = 0.003, I^2^ = 61%). Based on the sensitivity analysis, it was found that Redfern et al. 2019 and Demnitz et al. 2017 were the main contributors to heterogeneity, and after their exclusion, heterogeneity became insignificant (*p* = 0.142), resulting in a significant unchanged effect size of 0.3 (95% CI = 0.218–0.377, *p* = 0.000).

##### The Effect Size for the Correlation of Processing Speed and Balance

A meta-analysis of seven studies found a medium effect size of 0.287 (95% CI = 0.206–0.363, *p* < 0.000; Fig. 2), in favor of a positive association between processing speed and dynamic balance. The results suggest that older adults with faster processing speeds performed better on the dynamic tests. There was no significant heterogeneity (Q = 7.612, *p* = 0.268, I^2^ = 0.21).

A meta-analysis of four analysis results in four included studies found an overall small effect size of 0.166 (95% CI = 0.102–0.229, *p* < 0.000; Fig. 3), in favor of a positive association between processing speed measures and static balance. There was no significant heterogeneity (Q = 4.629, *p* = 0.201, I^2^ = 35).

##### The Effect Size for the Correlation of Episodic Memory and Balance

A meta-analysis of six studies found a very small effect size of 0.098 (95% CI = 0.063–0.131, *p* = 0.000; Fig. 2), in favor of a positive association between episodic memory measures and dynamic balance. In addition, the studies were not heterogeneous (Q = 4.880, *p* = 0.43, I^2^ = 0). We did not have enough studies for a meta-analysis of association between episodic memory and static balance.

##### The Effect Size for the Correlation of Global Cognition and Balance

A meta-analysis of nine studies revealed a medium effect size of 0.258 (95% CI = 0.134 to 0.370, *p* = 0.000; Fig. 2) in favor of a positive association between global cognition and dynamic balance. They were significantly heterogeneous (Q = 39.847, *p* = 0.000¸ I^2^ = 79). The result is stable after removing the studies one by one.

A meta-analysis of seven analysis results in nine included studies revealed a small effect size of 0.192 (95% CI = 0.113 to 0.268, *p* = 0.000; Fig. 3) in favor of a positive association between global cognition and static balance. This suggests that older adults with better performance on global cognition tests performed better on static balance measures. There was not significant heterogeneity (Q = 14.107, *p* = 0.079¸ I^2^ = 43).

#### Analyzing the Relationship Between Cognitive Domains and Dynamic Balance in Comparison to Cognitive Domains and Static Balance

Correlations between executive function, processing speed, and global cognition and dynamic balance had moderate effect sizes, whereas correlations between those and static balance had small effect sizes. The result was the same when the meta-analysis was done for each dynamic and static test with cognition which confirms the result.

## Discussion

The aims of this review are threefold: 1. to investigate the association between cognitive domains and balance in healthy older adults; 2. to pool the individual associations between each cognitive domain with static and dynamic balance to understand which cognitive domain is more sensitive to static or dynamic balance disturbances; 3. To Investigate whether this association is different from dynamic balance compared to static balance, and between different outcome measures of balance. To the best of our knowledge, this systematic review and meta-analysis is the first to compare the relationship between cognitive domains with static versus dynamic balance tasks, while the primary focus of previous systematic reviews has been on the broader association between physical and cognitive function [15••, 16•].

Regarding aim 1, the findings in this review showed a consistent positive association between cognitive domains (executive function, processing speed, and global cognition) and balance. The reviewed evidence shows that individuals with better balance perform better in assessments of global cognition, executive function, and processing speed. There have been some reports of non-significant findings, but the positive direction of all significant associations encouraged our conclusion. Regarding aim 2, the findings of this meta-analysis showed that the association was significant and consistent across all available cognitive domains. This consistency in findings suggests that the association between cognition and balance may not be exclusive to a single cognitive domain. However, the strength of this association was not equal for all cognitive domains, with executive function having the strongest and memory having the weakest association. Probably executive function and processing speed play more important roles in postural adjustment than episodic memory. Similarly, Demnitz et al. found an association between executive function and global cognition and postural balance in their meta-analysis. They reported a small effect size for this association, whereas we found a moderate effect size for dynamic balance and a small effect size for static balance in the present study [16•]. Compared to Demnitz et al.’s 2016 meta-analysis, which included only three studies, the current study included 32 studies. Furthermore, they considered balance as a general ability with no further sub-categorization, whereas in the current study, balance was categorized as static and dynamic subtypes.

The current review also showed a significant positive association of memory and processing speed with postural balance. These findings are consistent with previous studies [41, 42, 45, 57]. However, there is a discrepancy between these findings and those of Demnitz et al.’s study in 2017 [48]. The ceiling effect in this study could be one contributing factor to this disparity. Seventy-two percent of the participants completed the balance test at the ceiling for 72% of their participants. There is evidence that the relationship between cognition and balance manifests itself in more difficult activities [15••, 58, 59].

Regarding aim 3, this meta-analysis shows that dynamic balance has a moderate correlation with executive function, processing speed, and global cognition, while they have small correlation with static balance. Additionally, all balance tests (timed up and go, postural sway in dynamic and static conditions, and time in balance in single-leg stance position) were positively associated with cognition, with dynamic balance tests showing a moderate association, and static balance tests showing a small association. Interestingly, the correlation between cognitive domains and dynamic balance was found to be statistically greater than the correlation between cognitive domains and static balance. Although the brain structures responsible for controlling static and dynamic balance are the same, their differing contributions to each balance condition may explain these findings [60]. This suggests that the association between cognition and balance is task-specific and stronger in more complex balance tasks, such as dynamic balance tasks. The relationship between cognition and mobility is affected by task difficulty [59]. Cognitive inputs required for postural control vary with task complexity and the individual’s postural control abilities [4]. Dynamic balance tasks are more complex than static ones. Dynamic balance tasks, which involve continuous changes in the environment and acting forces, require greater cognitive involvement compared to static balance tasks [14••]. In addition, imaging studies in healthy older adults showed task-specific compensatory activation in several brain areas [61]. These findings further support the notion that the association between cognition and balance is task-specific and stronger in more complex balance tasks, such as dynamic balance tasks.

### Limitations

Two concerns were identified in terms of cognitive measures: inconsistency among studies in the tests used to measure cognitive domains and difficulty in accurately classifying cognitive domains. Two concerns were also identified in relation to balance outcome measures: variability in tests used to measure dynamic and static balance and the multifactorial nature of postural balance control. Factors affecting balance such as muscle strength or physical inactivity, may affect the relationship between cognition and balance. This review did not include participants with neurological conditions, and so cannot be generalized to those populations.

### Suggestions for future research

To shed light on the directionality of this relationship, more longitudinal studies are needed to assess whether balance or cognition is more likely to decline first over time. Further research into the mechanisms underlying the association between cognition and balance, including studies that measure brain activity during different balance tasks, is recommended. It is advisable to explore the correlation between cognitive domains and balance in various cognitive disorders as well, as they may impact balance differently.

## Conclusion

In conclusion, as for aim 1, this systematic review shows a positive association between balance and cognitive domains (executive function, processing speed, memory, and global cognition) in healthy older adults. For aim 2, while balance and cognition are not exclusively linked by one cognitive domain, executive function shows the strongest association with balance while memory shows the weakest association. For aim 3, a comparison of the correlation between cognitive domains and static versus dynamic types of balance showed that the association between executive function, processing speed, and global cognition and dynamic balance was moderate, whereas it was small between these cognitive domains and static balance. In addition, the association between cognition and each type of dynamic balance test was moderate, while it was small for this association with each type of static balance test. Hence, the type of balance task appears to influence the relationship between cognition and balance. These findings have implications for assessment, treatment planning, fall prevention, functional training, cognitive–motor integration, and rehabilitation outcomes. It allows clinicians to prioritize incorporating cognitive domains such as executive function and processing speed tasks as a dual task with dynamic balance interventions to enhance their effectiveness.

### Supplementary Information

Below is the link to the electronic supplementary material.

Supplementary file1 (DOCX 987 KB)

## References

[1] Mitchell E, Walker R (2020). Global ageing: successes, challenges and opportunities. Br J Hosp Med.

[2] Almeida L, Meucci RD, Dumith SC. Prevalence of falls in elderly people: a population based study. Rev Assoc Med Bras (1992). 2019;65(11):1397–403. 10.1590/1806-9282.65.11.1397 31800903

[3] Hopkins J, Hill K, Jacques A, Burton E (2023). Prevalence, risk factors and effectiveness of falls prevention interventions for adults living with Mild Cognitive Impairment in the community: A systematic review and meta-analysis. Clin Rehabil.

[4] Horak FB, Macpherson JM. Postural orientation and equilibrium. In Comprehensive Physiology. 2011; pp 255–292. 10.1002/cphy.cp120107

[5] Montero-Odasso M, Verghese J, Beauchet O, Hausdorff JM (2012). Gait and cognition: a complementary approach to understanding brain function and the risk of falling. J Am Geriatr Soc.

[6] Rosenbloom MH, Schmahmann JD, Price BH (2012). The functional neuroanatomy of decision-making. J Neuropsychiatry Clin Neurosci.

[7] Kwag E, Zijlstra W (2022). Balance tasks requiring inhibitory control; a scoping review of studies in older adults. Gait Posture.

[8] Liu Y, Ma W, Li M, Han P, Cai M, Wang F (2021). Relationship Between Physical Performance and Mild Cognitive Impairment in Chinese Community-Dwelling Older Adults. Clin Interv Aging.

[9] Mansouri FA, Tanaka K, Buckley MJ (2009). Conflict-induced behavioural adjustment: a clue to the executive functions of the prefrontal cortex. Nat Rev Neurosci.

[10] Murman DL (2015). The impact of age on cognition. Semin Hear.

[11] Clouston SAP, Brewster P, Kuh D, Richards M, Cooper R, Hardy R (2013). The dynamic relationship between physical function and cognition in longitudinal aging cohorts. Epidemiol Rev.

[12] Ghai S, Ghai I, Effenberg AO (2017). Effects of dual tasks and dual-task training on postural stability: a systematic review and meta-analysis. Clin Interv Aging.

[13] • Stuhr C, Hughes CML, Stöckel T. Task-specific and variability-driven activation of cognitive control processes during motor performance. Sci Rep. 2018;8(1):10811. **This study explain well about that the cognitive control process for motor performances are task specific**. 10.1038/s41598-018-29007-3 PMC6050332 30018399

[14] •• Rizzato A, Paoli A, Andretta M, Vidorin F, Marcolin G. Are static and dynamic postural balance assessments two sides of the same coin? A cross-sectional study in the older adults. Front Physiol. 2021;12:681370. **This study explain well about the different cognitive demands of static and dynamic balance**. 10.3389/fphys.2021.681370 PMC8277194 34267673

[15] •• Heaw YC, Singh DKA, Tan MP, Kumar S. Bidirectional association between executive and physical functions among older adults: a systematic review. Australas J Ageing. 2022;41(1):20–41. **This review checks the relationship between executive function with physical function, one of the physical function component of this study was balance**. 10.1111/ajag.13008 34724301

[16] • Demnitz N, Esser P, Dawes H, Valkanova V, Johansen-Berg H, Ebmeier KP, et al. A systematic review and meta-analysis of cross-sectional studies examining the relationship between mobility and cognition in healthy older adults. Gait Posture. 2016;50:164–74. **This review and meta-analysis check the relationship between congnition with mobility, one of the mobility component of this study was balance**. 10.1016/j.gaitpost.2016.08.028 PMC5081060 27621086

[17] Page MJ, McKenzie JE, Bossuyt PM, Boutron I, Hoffmann TC, Mulrow CD (2021). The PRISMA 2020 statement: an updated guideline for reporting systematic reviews. BMJ.

[18] Wells GA, Wells G, Shea B, Shea B, O’Connell D, Peterson J, Welch, Losos M, Tugwell P, Ga SW, Zello GA, Petersen JA. The Newcastle-Ottawa scale (NOS) for assessing thequality of nonrandomised studies in meta-analyses. 2014.

[19] Downes MJ, Brennan ML, Williams HC, Dean RS (2016). Development of a critical appraisal tool to assess the quality of cross-sectional studies (AXIS). BMJ Open.

[20] Wang Y, Li C (2022). Differences between the formation of tourism purchase intention and the formation of actual behavior: a meta-analytic review. Tour Manag.

[21] Rupinski MT, Dunlap WP (1996). Approximating Pearson product-moment correlations from Kendall’s tau and Spearman’s rho. Educ Psychol Meas.

[22] Peterson RA, Brown SP (2005). On the use of beta coefficients in meta-analysis. J Appl Psychol.

[23] Borenstein M, Hedges LV, Higgins JPT, Rothstein HR (2009). Introduction to meta-analysis.

[24] Higgins JP, Thompson SG (2002). Quantifying heterogeneity in a meta-analysis. Stat Med.

[25] Higgins JPT, Thompson SG, Deeks JJ, Altman DG (2003). Measuring inconsistency in meta-analyses. BMJ.

[26] Hand DJ (2012). Understanding The new statistics: effect sizes, confidence intervals, and meta-analysis by Geoff Cumming. Int Stat Rev.

[27] Bergquist R, Weber M, Schwenk M, Ulseth S, Helbostad JL, Vereijken B (2019). Performance-based clinical tests of balance and muscle strength used in young seniors: a systematic literature review. BMC Geriatr.

[28] Harvey PD (2019). Domains of cognition and their assessment. Dialogues Clin Neurosci.

[29] Kang SJ, Kim BH, Lee H, Wang J (2022). Association among cognitive function, physical fitness, and health status in older women. J Exerc Rehabil.

[30] Jovanović S, Stojanović Jovanović B, Pavlović A, Milosevic R, Pavlovic D. Cognitive ability and motor performances in the elderly. Vojnosanitetski pregled. 2020;79:143–143. 10.2298/VSP200812143J

[31] de Oliveira Matos F, Vido A, Garcia WF, Lopes WA, Pereira A. A neurovisceral integrative study on cognition, heart rate variability, and fitness in the elderly. Front Aging Neurosci 12. 2020. 10.3389/fnagi.2020.00051 10.3389/fnagi.2020.00051 PMC7068733 32210785

[32] Netz Y, Zeev A, Dunsky A (2018). Postural control and posture-unrelated attention control in advanced age-An exploratory study. Maturitas.

[33] Kose Y, Ikenaga M, Yamada Y, Morimura K, Takeda N, Ouma S (2016). Timed Up and Go test, atrophy of medial temporal areas and cognitive functions in community-dwelling older adults with normal cognition and mild cognitive impairment. Exp Gerontol.

[34] Blackwood Pt PDGCSCJ, Shubert T, Fogarty K, Chase C. Relationships Between Performance on Assessments of Executive Function and Fall Risk Screening Measures in Community-Dwelling Older Adults. J Geriat Phys Ther. 2001;2015;39. 10.1519/JPT.0000000000000056 26050194

[35] Kawagoe T, Suzuki M, Nishiguchi S, Abe N, Otsuka Y, Nakai R (2015). Brain activation during visual working memory correlates with behavioral mobility performance in older adults. Front Aging Neurosci.

[36] Berryman N, Bherer L, Nadeau S, Lauzière S, Lehr L, Bobeuf F (2013). Executive functions, physical fitness and mobility in well-functioning older adults. Exp Gerontol.

[37] Herman T, Giladi N, Hausdorff JM (2011). Properties of the ‘timed up and go’ test: more than meets the eye. Gerontology.

[38] Hirota C, Watanabe M, Sun W, Tanimoto Y, Kono R, Takasaki K (2010). Association between the Trail Making Test and physical performance in elderly Japanese. Geriat Gerontol Int.

[39] Won H, Singh DKA, Din NC, Badrasawi MM, Manaf ZA, Tan ST (2014). Relationship between physical performance and cognitive performance measures among community-dwelling older adults. Clin Epidemiol.

[40] Tsutsumimoto K, Doi T, Misu S, Ono R, Hirata S (2013). Can the Ordered Multi-Stepping Over Hoop test be useful for predicting fallers among older people? A preliminary 1 year cohort study. Aging Clin Exp Res.

[41] Redfern MS, Chambers AJ, Sparto PJ, Furman JM, Jennings JR (2019). Inhibition and decision-processing speed are associated with performance on dynamic posturography in older adults. Exp Brain Res.

[42] Redfern MS, Jennings JR, Mendelson D, Nebes RD (2009). Perceptual inhibition is associated with sensory integration in standing postural control among older adults. J Gerontol Ser B Psychol Sci Soc Sci.

[43] van Iersel MB, Kessels RP, Bloem BR, Verbeek AL, OldeRikkert MG (2008). Executive functions are associated with gait and balance in community-living elderly people. J Gerontol A Biol Sci Med Sci.

[44] Rabbitt PM, Scott M, Thacker N, Lowe C, Horan M, Pendleton N (2006). Balance marks cognitive changes in old age because it reflects global brain atrophy and cerebro-arterial blood-flow. Neuropsychologia.

[45] Zettel-Watson L, Suen M, Wehbe L, Rutledge DN, Cherry BJ (2017). Aging well: Processing speed inhibition and working memory related to balance and aerobic endurance. Geriat Gerontol Int.

[46] Muir-Hunter SW, Clark J, McLean S, Pedlow S, Van Hemmen A, Montero Odasso M (2014). Identifying balance and fall risk in community-dwelling older women: the effect of executive function on postural control. Physiother Can Physiother Can.

[47] Boolani A, Martin R, Goodwin A, Avolio A, Sur S, Lee Smith M, et al. Associations for tasks requiring single stimulus and working memory with different aspects of gait and posture: an exploratory study. Int J Rehab Res. 42(2):160–7. 10.1097/MRR.0000000000000347 30882529

[48] Demnitz N, Zsoldos E, Mahmood A, Mackay CE, Kivimäki M, Singh-Manoux A (2017). Associations between mobility, cognition, and brain structure in healthy older adults. Front Aging Neurosci.

[49] Bruce-Keller AJ, Brouillette RM, Tudor-Locke C, Foil HC, Gahan WP, Nye DM (2012). Relationship between cognitive domains, physical performance, and gait in elderly and demented subjects. J Alzheimers Dis.

[50] Rosano C, Simonsick EM, Harris TB, Kritchevsky SB, Brach J, Visser M (2005). Association between physical and cognitive function in healthy elderly: the health, aging and body composition study. Neuroepidemiology.

[51] Zhao X, Huang H, Du C (2022). Association of physical fitness with cognitive function in the community-dwelling older adults. BMC Geriatr.

[52] Abe T, Soma Y, Kitano N, Jindo T, Sato A, Tsunoda K (2017). Change in hand dexterity and habitual gait speed reflects cognitive decline over time in healthy older adults: a longitudinal study. J Phys Ther Sci.

[53] Kwan MM-S, Lin S-I, Chen C-H, Close JCT, Lord SR. Sensorimotor function, balance abilities and pain influence Timed Up and Go performance in older community-living people. Aging Clin Exp Res. 2011;23(3):196–201. 10.1007/BF03324960 21993166

[54] Woo MT, Davids K, Liukkonen J, Chow JY, Jaakkola T (2017). Falls, Cognitive Function, and Balance Profiles of Singapore Community-Dwelling Elderly Individuals: Key Risk Factors. Geriatr Orthop Surg Rehabil.

[55] Imaoka Y, Hauri L, Flury A, de Bruin ED (2022). Linking cognitive functioning and postural balance control through virtual reality environmental manipulations. Front Aging Neurosci.

[56] Goto S, Sasaki A, Takahashi I, Mitsuhashi Y, Nakaji S, Matsubara A. Relationship between cognitive function and balance in a community-dwelling population in Japan. Acta Oto-Laryngol. 138(5):471–4. 10.1080/00016489.2017.1408142 29205084

[57] Gatto NM, Garcia-Cano J, Irani C, Liu T, Arakaki C, Fraser G (2020). Observed Physical Function Is Associated With Better Cognition Among Elderly Adults: The Adventist Health Study-2. Am J Alzheimers Dis Other Demen.

[58] Demnitz N, Hogan DB, Dawes H, Johansen-Berg H, Ebmeier KP, Poulin MJ (2018). Cognition and mobility show a global association in middle- and late-adulthood: analyses from the Canadian longitudinal study on aging. Gait Posture.

[59] Poldrack RA, Sabb FW, Foerde K, Tom SM, Asarnow RF, Bookheimer SY (2005). The neural correlates of motor skill automaticity. J Neurosci.

[60] Takakusaki K (2013). Neurophysiology of gait: from the spinal cord to the frontal lobe. Mov Disord.

[61] Huang CM, Polk TA, Goh JO, Park DC (2012). Both left and right posterior parietal activations contribute to compensatory processes in normal aging. Neuropsychologia.

