# Supplementary material for: The Association Between Cognitive Domains and Postural Balance among Healthy Older Adults: A Systematic Review of Literature and Meta-Analysis
Source: Curr Neurol Neurosci Rep. 2023 Oct 19;23(11):681–93. doi: 10.1007/s11910-023-01305-y (PMC10673728; doi:10.1007/s11910-023-01305-y)
Supplement: Supplementary file 1 — Supplementary file1 (DOCX 987 KB) [file 11910_2023_1305_MOESM1_ESM.docx]

**Appendix A:**

**Search strategy and MeSH**

1. Postural balance.ab,kw,ti.
2. Poatural Sway.ab,kw,ti.
3. Equilbrium.ab,kw,ti.
4. Mobility ab,kw,ti.
5. Physical function ab,kw,ti.
6. 1 OR 2 OR 3 OR 4 OR 5
7. Cognition.ab,kw,ti.
8. Cognitive domains ab,kw,ti.
9. Executive function.ab,kw,ti.
10. Processing speed.ab,kw,ti.
11. Memory.ab,kw,ti.
12. Attention.ab,kw,ti.
13. Language.ab,kw,ti.
14. Mental.ab,kw,ti.
15. 7 OR 8 OR 9 OR 10 OR 11 OR 12 OR 13 OR 14
16. Association.ab,kw,ti.
17. Correlation.ab,kw,ti.
18. Relationship.ab,kw,ti.
19. 16 OR 17 OR 18
20. (1 OR 2 OR 3 OR 4 OR 5) AND (7 OR 8 OR 9 OR 10 OR 11 OR 12 OR 13 OR 14) AND (16 OR 17 OR 18).


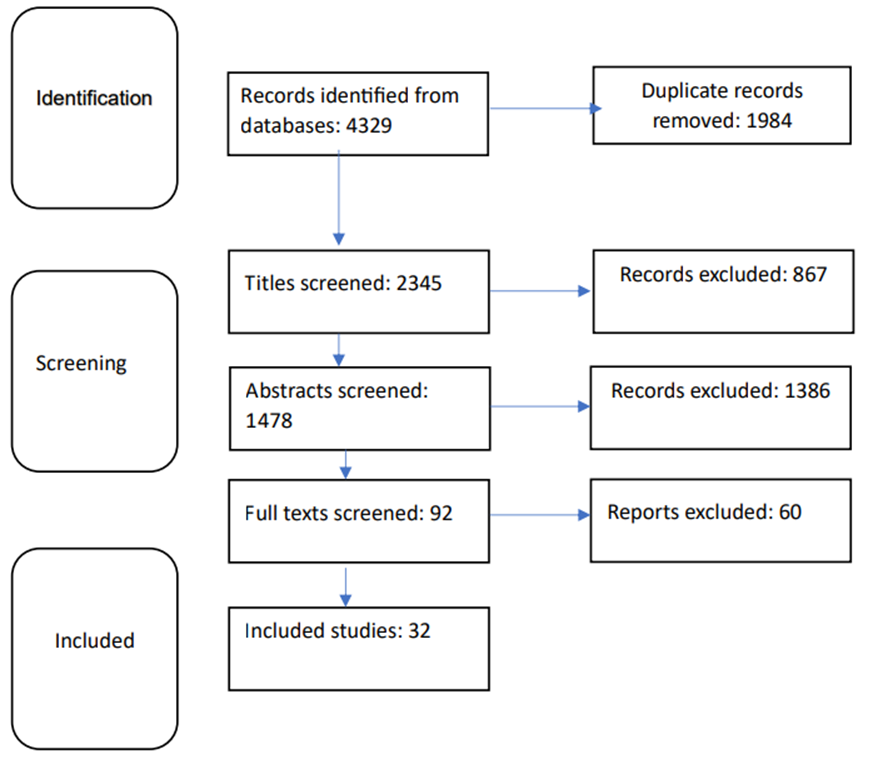


Figure 1. Flowchart for the process of literature search


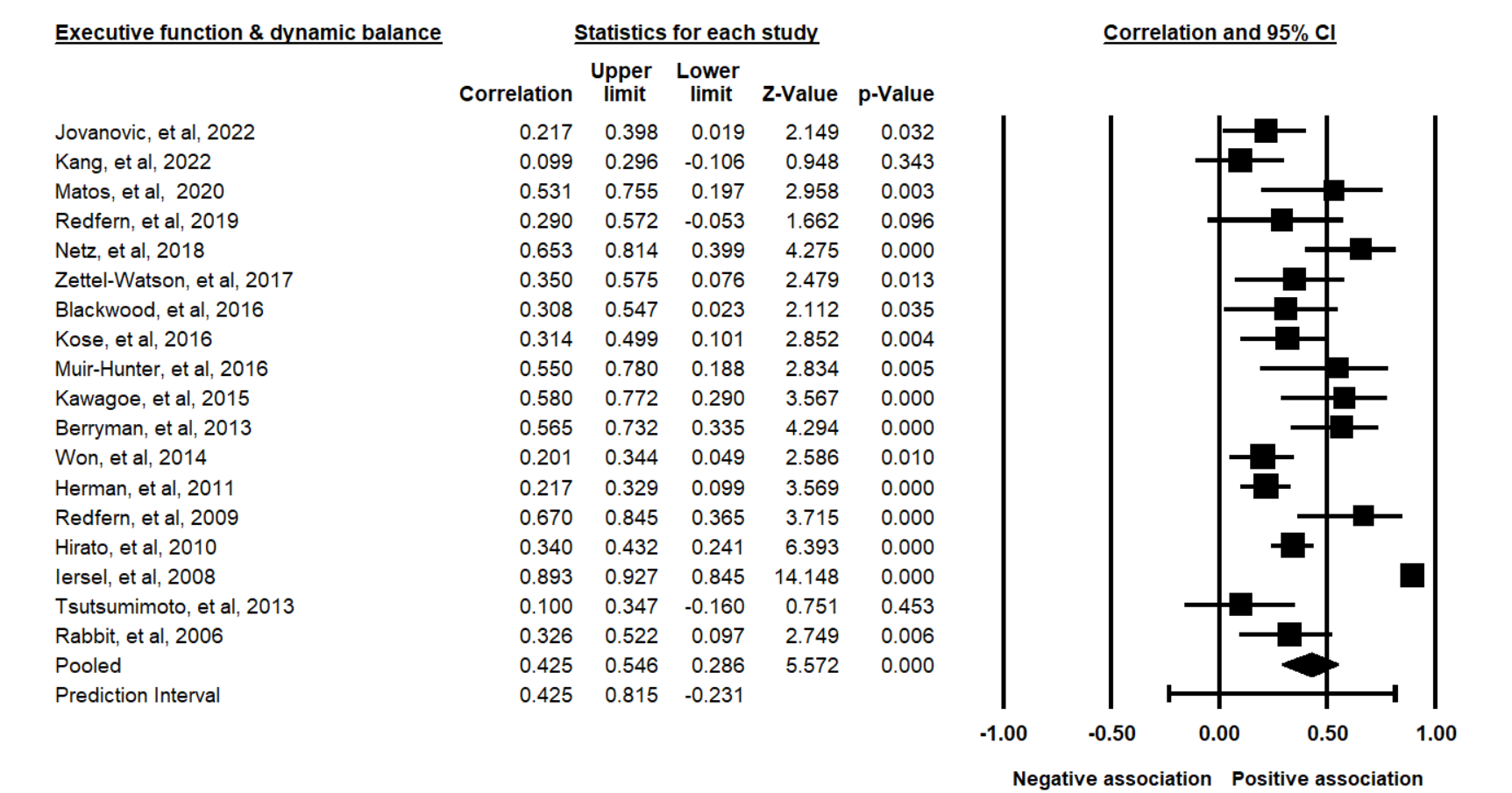


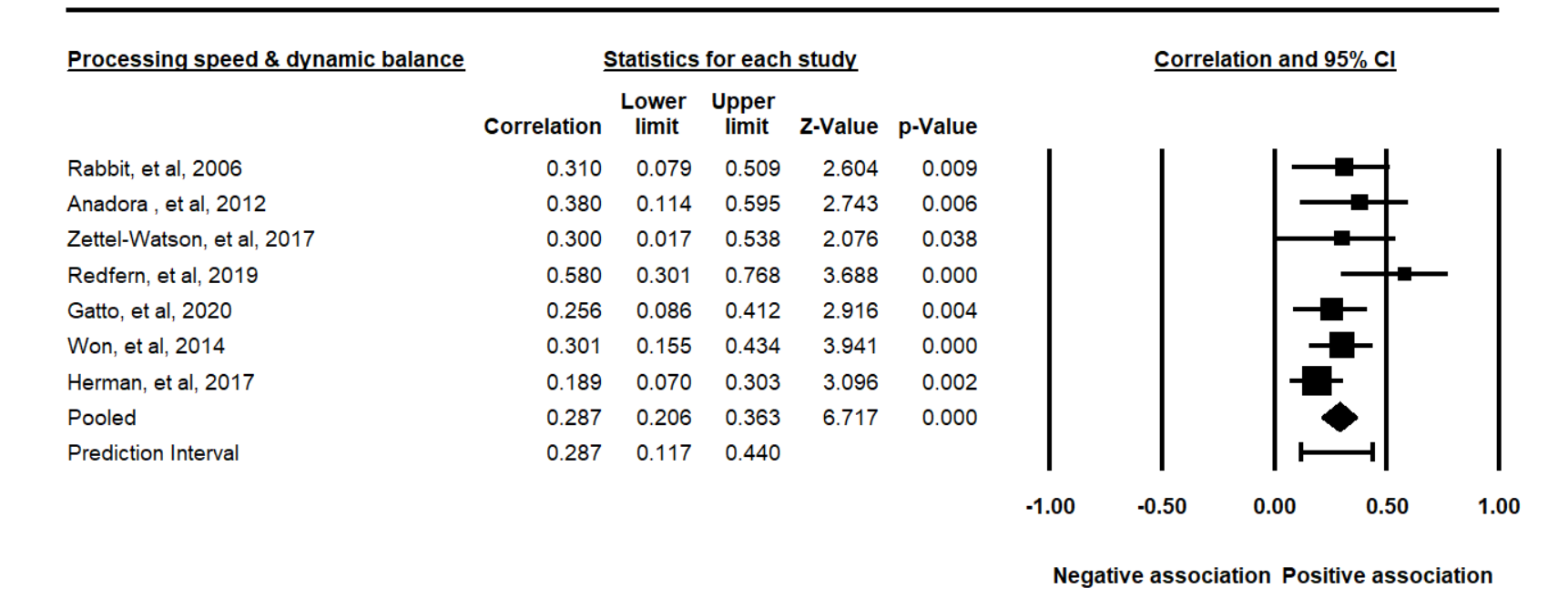


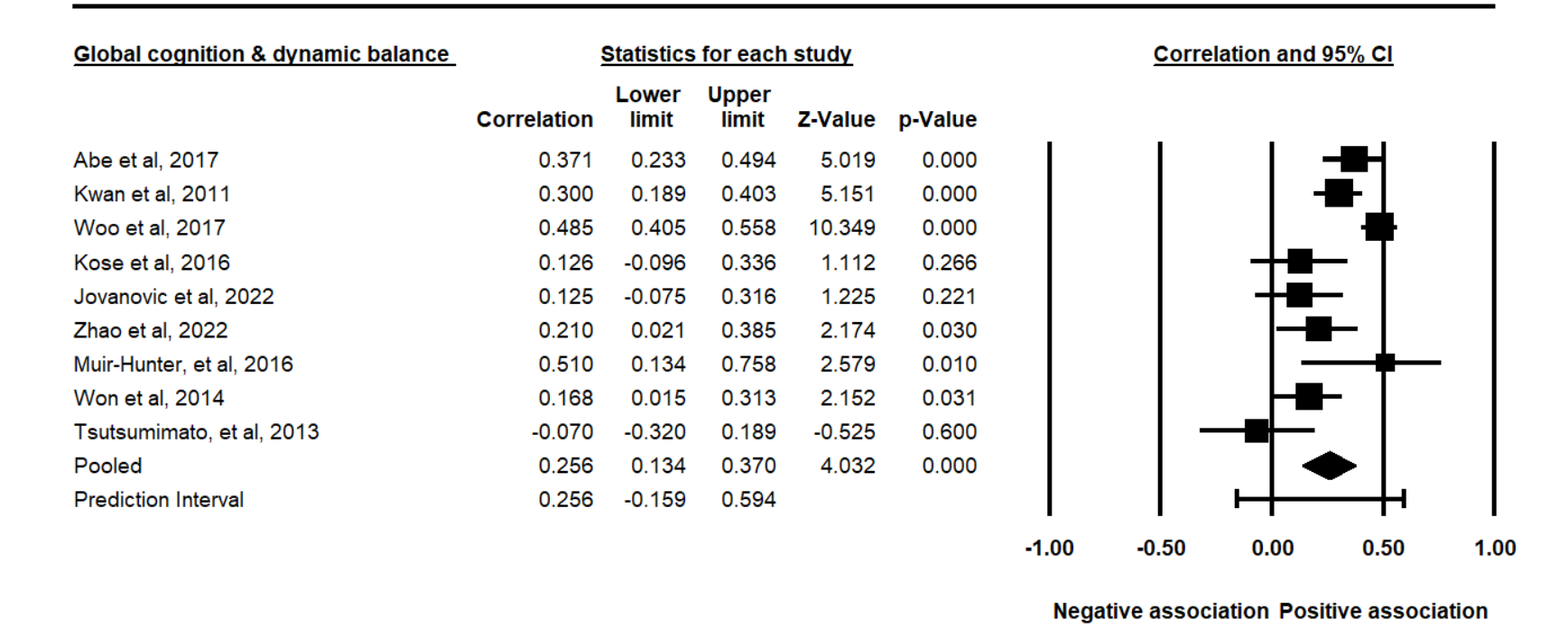


Figure 2. Statistical summary and forest plot of effect sizes for the association of executive function, processing speed, global cognition, and memory with dynamic balance


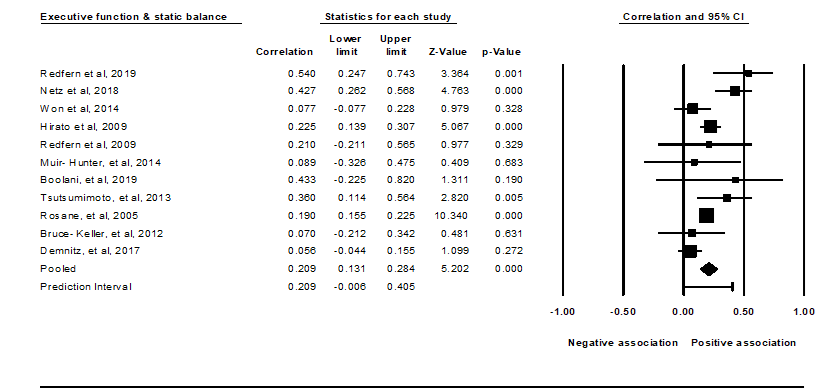

Figure 3. Statistical summary and forest plot of effect sizes for the association of executive function, processing speed, and global cognition with static balance

**Table 1:** Characteristics of the relationship between measures of processing speed, memory and dynamic/static balance.

| First Author | | Number of participants | Mean Age | | | % Female | | Balance  Task | Processing speed | Association |
| --- | --- | --- | --- | --- | --- | --- | --- | --- | --- | --- |
| **Processing speed and dynamic balance** | | | | | | | | | | |
| **Gatto, et al, (42)**  **2020** | | 127 | 74.9 + 7.9 | | | 57.5% | | TUG | Coding subtest of WAISNSIV, Stroop, and TMT | S  0.432 |
| **Herman, et al, (28)**  **2011** | | 265 | 76.4 | | | 58% | | TUG | Digit Span Test | S  r: 0.189 |
| **Redfern, et al, (32)**  **2019** | | 34 | 76.0±  4 | | | 61.7% | | Postural sway | MAPIT battery, Choice Reaction Time | S  r: 0.58 |
| **Zettel-Watson, et al, (36) 2017** | | 48 | 69.5±  8.1 | | | 64% | | FABS | Digit Symbol Substitution | S  r: 0.29 |
| **Won, et al,(30)**  **2014** | | 164 | 66 ± 4.6 | | | 66.5% | | FRT | Digit Symbol Test | S  r: 0.301 |
| Rabbit, et al, ^(35)^  2006 | | 69 | 73.2± 8.1 | | | 57.97% | | TBT | Digit Coding | S  r: 0.31 |
| Sprague, et al, (43)  2019 | | 2783 | 73.6±  5.7 | | | 73.60% | | Turn 360 | Useful Field of View | S  0.060 |
| **Processing speed and static balance** | | | | | | | | | | |
| Redfern, et al, (32)  2019 | | 34 | 76.0±  4 | | | 61.7% | | Postural sway | MAPIT battery, Choice Reaction Time | S  r: 0.36 |
| **Won, et al, (30)**  **2014** | | 164 | 66 ± 4.6 | | | 66.5% | | Postural Sway | Digit Span Test | NS  r: 0.052 |
| Demnitz, et al, (39)  2017 | | 387 | 69.0 ± 5.1 | | | 19% | | SLS | Digit span | S  r: 0.151 |
| Bruce- Keller, et al,(40)  2012 | | 50 | 74.2±  7.8 | | | 42% | | Balance SPPB | Digit Symbol Test | S  r: 0.29 |
| First Author | Number of participants | | Mean Age | | % Female | | | Balance  Task | Memory | Association |
| **Episodic Memory and dynamic balance** | | | | | | | | | | |
| **Kang et al, (20)**  **2022** | 94 | | 77.6±  5.3 | | 100% | | | TUG | Seoul Neuropsychological Screening Battery | NS  r: 0.304 |
| Jovanovic, et al,(21) 2022 | 98 | | 68.5 | | 83.6% | | | TUG | Delayed recal the 5-word list | NS  r: 0.076 |
| Sprague, et al, (43)  2019 | 2783 | | 73.6±  5.7 | | 73.60% | | | Turn 360 | Hopkins Verbal Learning Test | S  r: 0.1 |
| Kose, et al, (24)  2016 | 80 | | 75.7± 5.8 | | 45% | | | TUG | Wechsler Memory, logical memory I | NS  r: 0.073 |
| **Won, et al, (30)**  **2014** | 164 | | 66 ± 4.6 | | 66.5% | | | FRT | Rey Auditory Verbal Learning Test | NS  r: 0.142 |
| Rabbit, et al, (35)  2006 | 69 | | 73.2± 8.1 | | 57.97% | | | TBT | Memory for Object and Location | S  r: 0.26 |
| **Episodic Memory and static balance** | | | | | | | | | | |
| Demnitz, et al, (39)  2017 | 387 | | 69.0 ± 5.1 | 19% | | | SLS | | Hopkins Verbal Learning Test | S  r: 0.063 |
| **Won, et al, (30)**  **2014** | 164 | | 66 ± 4.6 | 66.5% | | | Postural sway | | Rey Auditory Verbal Learning Test | NS  r: 0.151 |

Table 1: No: Number of participants, M: male, F: Female, Number: reference of the study; TUG: Timed Up and Go Test; FRT: Functional Reach Test; TBT: Tinetti Balance Test; BBT: Berg Balance Test; FABS: Fullerton Advanced Balance Score; SPBB: Balance Score on the Short Physical Performance Battery. SLS: Single leg stance time, mCTCIB: Modified Clinical Test of Sensory Interaction on Balance. NS: Non-significant, S: Significant. r: correlation. Bolds are studies which had MMSE score>24 as inclusion criteria.
